# Platelet-rich plasma (PRP) therapy for knee arthritis: a feasibility study in primary care

**DOI:** 10.1186/s40814-018-0288-2

**Published:** 2018-07-04

**Authors:** Liam G. Glynn, Alaa Mustafa, Monica Casey, Janusz Krawczyk, Jeanete Blom, Rose Galvin, Ailish Hannigan, Colum P. Dunne, Andrew W. Murphy, Christian Mallen

**Affiliations:** 1 Graduate Entry Medical School and Health Research Institute, University of Limerick, Limerick, Ireland; 2 Department of Haematology, University College Hospital Galway, Galway, Ireland; 3 Leiden University, Rapenburg 70, 2311 EZ Leiden, Holland Netherlands; 4 Department of Clinical Therapies, Health Research Institute, University of Limerick, Limerick, Ireland; 5 HRB Primary Care Clinical Trial Network Ireland, School of Medicine, National University of Ireland, Galway, Ireland; 6 Research Institute for Primary Care and Health Sciences, Keele University, Staffordshire, UK

**Keywords:** Chronic disease, Musculoskeletal/connective tissue disorders, Orthopaedics, Therapeutic injection, Osteoarthritis of the knee, Community medicine, Managed care

## Abstract

**Background:**

Platelet-rich plasma (PRP) is a concentrate of autologous blood growth factors which has been shown to provide some symptomatic relief in early osteoarthritis (OA) of the knee. The objective of this study was to test the feasibility and efficacy potential of platelet rich plasma (PRP) in primary care.

**Methods:**

Feasibility study to assess safety of the intervention procedures and assess primary and secondary outcome measures. Consecutive patients presenting with symptomatic knee OA were recruited in a primary care setting in Ireland. All participants received three injections of PRP 4 weeks apart. The following self-reported clinical outcomes were evaluated before and after therapy (4 months): Pain and disability (ICOAP questionnaire); health utility (EUROQol); adverse events; patient satisfaction and goal-orientated outcomes.

**Results:**

Seventeen potential patients were identified of whom 14 were eligible to participate. Twelve consented and completed the intervention and all outcome measures. There were no losses to follow-up. One patient reported pain and stiffness for 2 days after the first injection but did complete the study. No growth was detected from nine consecutive samples sent for microbiology analysis. Changes in constant, intermittent and total pain scores were reported; pain fully resolved in two patients. In addition, health utility, patient satisfaction and goal-orientated outcomes also demonstrated improvement.

**Conclusions:**

Platelet-rich plasma therapy is a simple and minimally invasive intervention which is feasible to deliver in primary care to treat osteoarthritis of the knee joint. Well-designed randomised controlled trials are needed to measure outcomes, durability of effect and cost effectiveness.

## Key messages

- Platelet-rich plasma (PRP) is a concentrate of autologous blood growth factors.
- PRP has been shown to provide some symptomatic relief in knee osteoarthritis.
- To date, this intervention has been largely delivered in hospital settings.
- This study has shown that it is feasible to deliver PRP therapy in primary care.
- This therapy appears to have minimal associated adverse events.
- This therapy appears to be associated with improvements in patient outcomes.

## Background

Osteoarthritis (OA) is a leading cause of disability and doubles the number of visits to primary care practitioners for those with the condition in comparison to those without [1]. OA affects the knee more often than any other joint [2]. With the ageing of the population and the growing obesity epidemic, the number of surgical procedures for knee OA will increase dramatically in the coming years, of which knee replacement is the most costly to the health care system and burdensome for the patient. Other treatment options for OA of the knee would be of great value.

The joint destruction arising from OA occurs as a result of an imbalance in the equilibrium between the breakdown and repair of the joint tissue while a combination of cellular changes and biomechanical stresses causes several secondary changes in the joint itself. Recent research has identified a number of key biochemical pathways that could be targeted therapeutically through biological intervention [3]. Platelet-rich plasma (PRP) is one such intervention. PRP is an autologous concentration of human platelets in a small volume of plasma, where the platelet concentration is higher (typically up to five times higher) than the normal platelet concentration in a healthy person’s blood. Emerging evidence suggests PRP has the potential to have a regenerative effect on certain body tissues, in addition to the main role platelets play in haemostasis [4]. PRP has been shown to provide some symptomatic relief in early OA of the knee and to be at least as effective as intra-articular hyaluronic acid and steroid injections for symptom control [5]. This therapy is a minimally invasive intervention which could be used to enhance tissue regeneration. PRP contains alpha granules, in which about 70% of their growth factors will be secreted in the first 10 min, and almost all the stored amount will be released in the first hour [6]. These growth factors activate some of the cells which are responsible for tissue healing and bone and cartilage regeneration [7]. As PRP is an autologous blood product, there is no risk of immunological reactions and disease transfer, but as with any injection procedure, there will be some possibility of a local anaesthesia reaction, infection and bleeding [8]. The current study is designed to better understand this new therapy and to see if it is feasible to carry out this therapy in primary care in order to potentially avoid expensive hospital visits and interventions such as joint replacement. Current NICE guidelines confirm that PRP injections for knee osteoarthritis raise no major safety concerns, but the evidence on efficacy is inadequate and requires further research [9]. The aim of this study is to test the feasibility and efficacy potential of platelet-rich plasma (PRP) in primary care to treat degenerative lesion of articular cartilage of the knee in order to prepare for a phase II pilot randomised controlled trial of this intervention in primary care according to the Medical Research Council evaluation framework [10]. The specific objectives were to determine (1) trial feasibility, including recruitment, retention and assessment of outcome measures and (2) intervention feasibility, including intervention fidelity, attendance, acceptability to participants and potential of primary care to deliver the intervention.

## Methods

### Participants and setting

**Fig. 1:**
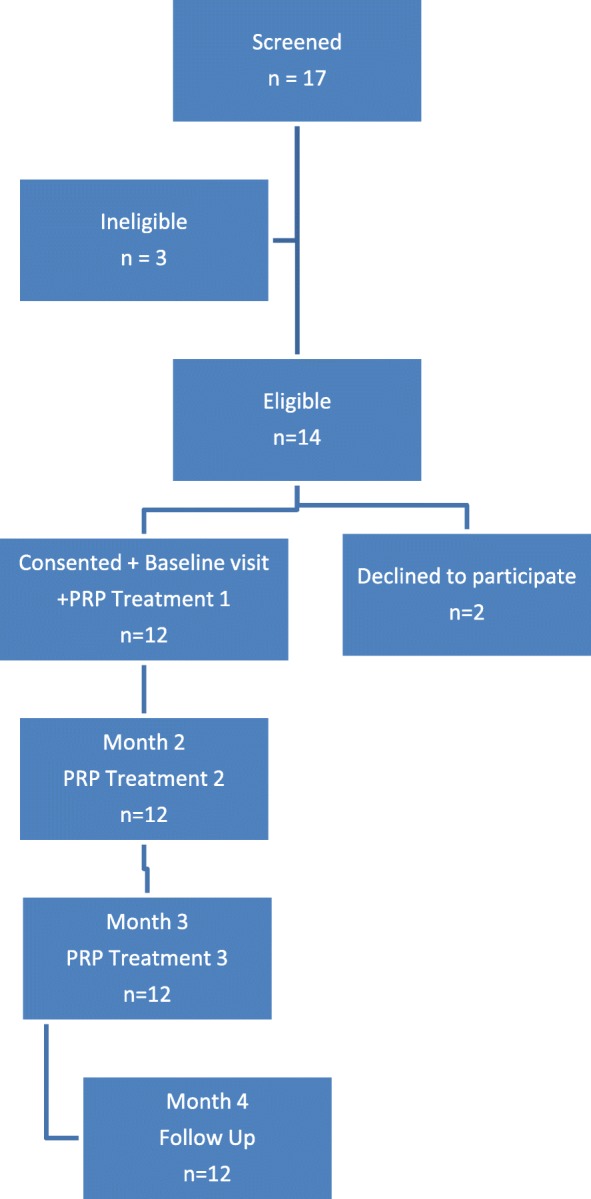
Flow diagram of the study

This study took place in the West of Ireland, and participants were recruited and followed up through the North Clare Primary Care Team, a rural-based primary care team covering an economically diverse but predominantly Caucasian population of approximately 8000 individuals [11]. Consecutive patients presenting to primary care with acute symptoms on a background of at least a 3-month history of chronic pain or swelling of the knee with radiologically confirmed (X-ray or MRI) osteoarthritis of the knee joint were screened for inclusion in the study. The following exclusion criteria were applied: cognitive impairment, acute mental or physical illness; unstable medical conditions; under 18 years of age; pregnancy and breastfeeding; coagulation disorders; immunosuppression; pending legal action pertaining to knee pain; cortisone injection within 6 weeks; use of NSAIDS 1 week beforehand who were unwilling to stop medication; inflammatory arthritis; previous infection of the knee joint; knee surgery within 3 months; active infection or malignancy; patients on Warfarin with an INR > 3. At the first contact, information about the study was provided to potential participants by their family doctor and they were invited to attend a preliminary screening meeting at their primary care centre with the study investigators. If eligible, informed consent was obtained and participants were assigned a code. All participants then completed a baseline assessment, including confirmation of diagnosis of knee arthritis and signed consent. Baseline visit at their primary care centre was then arranged at which they completed baseline questionnaires on outcome measures and then received their first PRP treatment. The treatment protocol used was in line with international best practice and consisted of three injections separated by 4 weeks each. Follow-up outcome measures were collected 4 weeks after the last PRP treatment (Fig. 1).

### Intervention

The preparation of the PRP begins by taking specific volume of autologous blood from the patient, through a syringe containing an anticoagulant. This sample is then centrifugated into two layers: an inferior layer containing erythrocytes and a superior layer consisting plasma, in which the platelet layer will be isolated and injected into the patient’s knee under a sterile environment [6]. The preparation of PRP for the study focussed on the following key processes taking into account the latest European Guidelines on the use of platelet-rich plasma [12]: Achieving a platelet concentration above baseline; use of anticoagulation; and addition of exogenous activation. In accordance with the guidelines, venepuncture, centrifugation and injection were all being carried out within a single procedure by a healthcare professional. No blood products were frozen or stored. The centrifuge used in the procedure was located in the same room as the procedure took place and was not used for spinning any other samples during the procedure.

Recommendations for the standardisation of the PRP procedure have been well described and were carefully adhered to in order to achieve appropriate platelet concentrations [13]. Fresh blood was drawn from the intervention patient and prepared directly before each injection of PRP. In order to check sterility, small volumes (2.8 ml) of prepared PRP were sent for microbiology testing to ensure complete sterility. Venipuncture can result in contamination of collected blood [14]. To provide confidence that such contamination had not occurred, or that PRP had not become contaminated during processing, microbiology analysis involved serial dilution and incubation of samples aerobically and anaerobically at 37 °C on plate count agar, which is a growth medium suitable for detection of skin-derived microorganisms. Blood was drawn into a closed system of anticoagulant vacutainer tubes containing 3.8% sodium citrate. Each tube contained a maximum volume of 2.8 ml so eight tubes were drawn from each participant. This system has a needle of 21 gauge for venepuncture to minimise any platelet activation. These tubes then underwent the first spin cycle in a Labofuge 200 ™ centrifuge manufactured by Heraeus and owned and calibrated by the Clinical Research Facility Galway (CRFG). This spin cycle was at 900*g* for 5 min. For this machine, this translates as a speed of 2900 revolutions per minute (rpm) based on the following calculation: *g* = 1.12 × radius of centrifuge × (rpm/1000)^2^. The tubes were then removed from the centrifuge and the plasma supernatant aspirated immediately (to prevent diffusion), using sterile technique, into a closed sterile container for the second spin which was at 1000*g* for 10 min. For this machine, this translates as a speed of 3100 rpm based on the same calculation given above.

After the second spin, the lower 1/3 of plasma containing the concentrated platelet solution was aspirated again using sterile technique, into a closed system containing 10% Ca-chloride (Ca^2+^ = 0.22 mEq) for platelet activation. The final volume for injection was 4–5 ml. All injections were performed by the same physician (LG) who had received training in and been carrying out knee arthrocentesis as part of routine clinical practice. Having prepared the skin of the participant with iodine solution and using a ‘no touch’ aseptic technique, the activated PRP was injected into the knee joint using a superomedial approach. Ethinyl chloride spray was applied to the skin just prior to injection to provide topical anaesthesia.

#### Outcome measures

We piloted the planned outcome measures to be used in the main trial, to ensure that they were acceptable and comprehensible to patient, and that our methods of administration were feasible and reliable. Self-reported clinical outcomes were evaluated before and after therapy (4 months). All self-report measures used were in previously validated formats. The primary and secondary outcome measures were chosen in accordance with OMERACT guidelines and were the following: Constant, intermittent and total pain scores were measured using the ICOAP questionnaire for knee arthritis; health utility was measured using EUROQol (EQ-5D-3L and EQ-VAS); complications and all adverse events were measured using adverse event forms at 8 and 16 weeks; overall patient satisfaction was measured using a Likert scale from 1 to 5 where 1 is very dissatisfied and 5 is very satisfied; and finally, we elicited patient goal-orientated outcomes. This final outcome measure was chosen as in a disease such as OA which more often than not exists in the context of multimorbidity; we felt it was particularly important to ask patients what they wanted from the intervention rather than concentrate only on established and validated outcome measures [15]. Patients were asked to describe their goals and also whether these goals had been achieved at follow-up (Not achieved, Partially achieved, Fully achieved).

### Statistical analysis

A formal sample size for this feasibility study was not calculated [16]. Baseline data was summarised using suitable numerical summaries and graphical techniques. Adverse events were reported individually at each time point. The EQ-5D-3L index value for health status was calculated for each patient with reference to a general population survey from the UK [17]. The distribution of changes from baseline to follow-up for each numeric outcome variable was tested for normality, and paired samples *t* tests were used for normally distributed changes. Mean changes with 95% confidence intervals (CI) are reported. A 5% level of significance was used for all tests. All statistical analyses were performed using the software package SPSS (version 24.0).

## Results

### Recruitment and baseline characteristics of trial participants

Of the 17 patients screened for the study, 14 were eligible to participate and 12 of these agreed to participate and were consented (Fig. 1). Of the three patients who were not eligible to participate, two had a cortisone injection within 6 weeks and one had inflammatory arthritis. The 12 participants that were eligible and consented had a mean age (SD) of 72.6 (10.4) years and mean BMI (SD) of 31.8 (4.6), and seven (58%) were male. Other baseline clinical characteristics are described in Table 1. Goal-orientated outcomes were identified by 92% (11/12) of the group. Of this group, the most common goal defined was to be pain free (6/12), followed by walking normally without aid (2/12). Other goals included decreasing knee stiffness (1/12), prevention of knee replacement (1/12) and being able to dance and garden again (1/12). The flow chart in Fig. 1 represents the movement of participants through the stages of the study. Of the 12 participants that were eligible and consented, all completed the full study protocol and follow-up at 4 months.

**Table 1:** Baseline characteristics of the participants (*n* = 12)

Baseline characteristics	
	Mean (SD); Range; Median
Age (years)	72.6 (10.4) years; 48–84 years; 73 years
Weight (kg)	89.3 (16.7) kg; 70-132 kg; 90 kg
^∫^Body mass index	31.8 (4.6); 25–42; 31.5
	Number of patients (%)
Male gender	7 (58)
Female gender	5 (42)
^∫^Body mass index categories	
BMI < 25	0 (0)
BMI 25–30	4 (33)
BMI > 30	8 (66)
^#^Medical card eligibility	10 (83)
Diagnosed by X-ray	10 (83)
Previous knee injury	8 (67)
Previous knee surgery	2 (17)

^∫^Body mass index is weight in kilogrammes divided by the square of the height in metres

^#^Ireland does not have universal registration with a general practitioner. Almost 45% of the population is registered through the Primary Care Reimbursement Service (PCRS) (http://www.hse.ie/eng/staff/PCRS/) with the remainder being described as private patients and able to see any general practitioner. All patients aged over 80 years and those below defined income levels (less than € 500 [£444] gross per week for a single person; € 900 [£798] gross per week for a couple) are registered with the PCRS and are described as ‘Medical Card’ eligible

### Outcome data

A total of 39 knee injections of PRP were carried out on 12 patients (One patient had both knees injected on each occasion). No patients reported adverse events at the time of injection. One patient (8%) had pain and stiffness after the injection for 2 days which responded to paracetamol. There were no other adverse events noted at 8 or 16 weeks. There was no evidence of microbial growth after 72 h incubation from the nine consecutive samples tested. In terms of responder status, at the end of 4 months, 58.3% (7/12) were very satisfied with the procedure, 33.3% (4/12) were satisfied and one patient (8.3%) was not satisfied. This patient reported the adverse event described above. In terms of goal-orientated outcomes, 45.5% (5/11) felt they had fully achieved their identified goal, 45.5% (5/11) felt they had partially achieved their goal and 9% (1/11) felt they had not achieved their goal either fully or partially. Again, it was this patient that reported the adverse event described above.

Baseline values, follow-up scores and change from baseline to follow-up is summarised in Table 2 for constant, intermittent and total pain scores. The reductions in pain scores (95% CI) for total, constant and intermittent pain were − 29.0 (− 39.1, − 18.9); − 28.8 (− 43.7, − 13.8); and − 29.2 (− 41.5, − 16.8), respectively.

**Table 2:** Outcomes measures at baseline, follow-up (4 months) and change from baseline (*n* = 12)

Outcome	Baseline Mean (SD) and range	Follow-up Mean (SD) and range	Mean change from baseline (95% CI)
*Total pain score	45.3 (17.38) 25.0–77.3	16.3 (17.28) 0–63.6	− 29.0 (− 39.1, − 18.9)
*Constant pain subscore	35.0 (27.96) 0–75.0	6.3 (16.39) 0–55.0	− 28.8 (−43.7, − 13.8)
*Intermittent pain subscore	53.8 (10.87) 41.7–79.2	24.7 (21.06) 0–70.8	− 29.2 (−41.5, − 16.8)
#EQ-5D-3L	0.45 (0.19) 0.08–0.62	0.77 (0.25) 0.08–1.00	+ 0.32 (0.18, 0.46)
#EQ-VAS	63.4 (9.10) 50–80	74.3 (17.72) 40–100	+ 10.8 (2.5, 19.2)

*A reduction in pain scores indicates improvement

#An increase in quality of life score indicates improvement

At baseline, eight (67%) patients reported being in constant pain (constant pain subscore > 0). This decreased to two patients reporting constant pain at follow-up. All 12 patients had total pain and intermittent pain scores greater than zero at baseline. Two patients reported that their pain was fully resolved (total pain score = 0) at follow-up.

Mean self-rating of health utility (EQ-VAS) increased from baseline to follow-up (mean change 10.8, 95% CI 2.5 to 19.2) (Table 2). Mean index values (EQ-5D-3L) also increased from baseline to follow-up (mean change 0.32, 95% CI 0.18 to 0.46) (Table 2).

## Discussion

### Summary

This study has demonstrated that it is feasible and safe to deliver PRP therapy in primary care for knee osteoarthritis. All patients completed the intervention and all outcome measures. There were no losses to follow-up. This therapy appears to be associated with significant improvements in pain, health utility, patient satisfaction and goal-orientated outcomes.

### Comparison with existing literature

Pilot and prospective studies investigating the clinical efficacy of intra-articular injections of PRP in patients with knee OA have demonstrated clinical improvement in self-reported pain and functional capacity with no major adverse effects [18]. In a recent related systematic review, conducted by our research team, we included six randomised controlled trials comparing the effectiveness of PRP to other intra-articular injections, exercise or analgesia for a minimum of 6 months. PRP injections were found to produce statistically significant improvements in overall WOMAC scores for patients with knee osteoarthritis up to 12 months after intervention [19]. The risk of adverse events in PRP-treated participants was not significantly increased in comparison with other knee osteoarthritis treatment options [19] These findings are consistent with much recently published research involving PRP as an intervention in knee OA [20]. The goal-oriented outcome approach used in this study has several advantages. It frames the discussion in terms of individually desired rather than universally applied health states; this approach simplifies decision making for patients with multiple conditions by focusing on outcomes that span conditions and aligning treatments toward common goals; goal-oriented care prompts patients to articulate which health states are important to them and their relative priority; and finally, if they know what health states are most desired, patients and clinicians can agree on steps that can be taken to achieve these goals and monitor progress in reaching them [15]. In essence, it allows the participant to co-design the outcomes based on their own individual preferences.

### Strengths and limitations

The strengths of this study are the clearly described standard operating procedure, the high completion rate for participants (100%) and the limited number of exclusion criteria. The relatively small number of potential participants that were excluded may help to facilitate implementation particularly with such an open recruitment strategy. However, this study also had a number of limitations. It was a small feasibility study conducted in a single country in a Caucasian population with short follow-up and without a control group.

### Implications for research and practice

The findings of our study are in keeping with other experimental studies of this nature [21, 22]; however, there is heterogeneity across studies with regard to the severity of the OA populations included, and the frequency, dose and duration of PRP interventions. In addition, the long-term outcomes of this form of therapy have not been established, and the duration of the expected benefit of PRP injections remains unclear, as most of the other studies investigate the persistence of the desirable effects up to 12 months post interventions but only a small number of studies has a follow-up period beyond that. Also, there is wide variety of PRP preparation protocols across different studies, for example, in terms of total number of absolute platelet and presence or absence of white blood cells, so consensus about the standardisation of PRP is needed [23]. Patient choice seems also to be an important factor as it has been shown that younger patients with earlier stage OA seem to be the most responsive subgroup to treatment [24]. The number of injections required is also not established with three injections separated by 4 weeks each being the most commonly used protocol, but a previous study has shown that a single injection of PRP is as effective as two injections [3]. The next obvious steps following on from this feasibility study are a phase II pilot randomised controlled trial in primary care to assess feasibility of trial methods followed by a full methodologically robust, randomised controlled trial to determine the effectiveness of this form of therapy, both for short- and long-term outcomes. In addition, the experience of patients following participation in this form of therapy warrants further consideration through the use of qualitative methods.

Our findings have a number of tentative clinical implications. These should be contextualised in the limitations of the study. Our results suggest that PRP is a safe and feasible therapy that can be delivered in the primary care setting. The arthrocentesis element of this intervention does require training and experience but is already within the scope of practice of many primary care physicians. However, adequate resourcing will be required to make this form of intervention widely available in primary care.

The main side-effect of PRP injection is pain at and around the injection site. Current evidence suggests that this pain is uncommon, responds to simple analgesics and may last for a few days. Finally, all injections were delivered by an experienced family doctor. However, there is scope to explore the role of musculoskeletal clinical specialist physiotherapists in the delivery of this treatment approach.

## Conclusions

Platelet-rich plasma therapy is a simple, low-cost and minimally invasive intervention which is feasible to deliver in primary care to treat degenerative lesions of articular cartilage of the knee. This therapy appears to have minimal associated adverse events and may have beneficial effects in terms of pain, health utility, patient satisfaction and goal-orientated outcomes. Further studies, particularly well-designed randomised controlled trials are needed to understand the mechanism of action, establish best practice, and measure outcomes and durability of effect.

## References

[1] Wright EA, Katz JN, Cisternas MG, Kessler CL, Wagenseller A, Losina E (2010). Impact of knee osteoarthritis on health care resource utilization in a US population-based national sample. Med Care.

[2] Duymus TM, Mutlu S, Dernek B, Komur B, Aydogmus S, Kesiktas FN (2017). Choice of intra-articular injection in treatment of knee osteoarthritis: platelet-rich plasma, hyaluronic acid or ozone options. Knee Surg Sports Traumatol Arthrosc.

[3] Patel S, Dhillon MS, Aggarwal S, Marwaha N, Jain A (2013). Treatment with platelet-rich plasma is more effective than placebo for knee osteoarthritis: a prospective, double-blind, randomized trial. Am J Sports Med.

[4] Lee KS, editor. Platelet-rich plasma injection. Seminars in musculoskeletal radiology. New York: Thieme Medical Publishers; 2013. 10.1055/s-0033-1333943 23487341

[5] Lana JFSD, Weglein A, Sampson SE, Vicente EF, Huber SC, Souza CV (2016). Randomized controlled trial comparing hyaluronic acid, platelet-rich plasma and the combination of both in the treatment of mild and moderate osteoarthritis of the knee. J Stem Cells Regen Med.

[6] Spakova T, Rosocha J, Lacko M, Harvanova D, Gharaibeh A (2012). Treatment of knee joint osteoarthritis with autologous platelet-rich plasma in comparison with hyaluronic acid. Am J Phys Med Rehabil.

[7] Say F, Gurler D, Yener K, Bulbul M, Malkoc M (2013). Platelet-rich plasma injection is more effective than hyaluronic acid in the treatment of knee osteoarthritis. Acta Chir Orthop Traumatol Cechoslov.

[8] Ko GD (2010). Platelet-rich plasma injection.

[9] National Institute for Clinical Excellence N. Platelet-rich plasma injections for osteoarthritis of the knee 2014. https://www.nice.org.uk/guidance/ipg491.

[10] Eldridge SM, Lancaster GA, Campbell MJ, Thabane L, Hopewell S, Coleman CL (2016). Defining feasibility and pilot studies in preparation for randomised controlled trials: development of a conceptual framework. PLoS One.

[11] Central Statistics Office (2012). Census 2011 preliminary report: central statistics office.

[12] Fiorentino S, Roffi A, Filardo G, Marcacci M, Kon E (2015). European definitions, current use, and EMA stance of platelet-rich plasma in sports medicine. J Knee Surg.

[13] Dhurat R, Sukesh M (2014). Principles and methods of preparation of platelet-rich plasma: a review and author's perspective. J Cutan Aesthet Surg.

[14] O'Connor C, Philip RK, Powell J, Slevin B, Quinn C, Power L (2016). Combined education and skin antisepsis intervention for persistently high blood-culture contamination rates in neonatal intensive care. J Hosp Infect.

[15] Reuben DB, Tinetti ME (2012). Goal-oriented patient care—an alternative health outcomes paradigm. N Engl J Med.

[16] Billingham SAM, Whitehead AL, Julious SA (2013). An audit of sample sizes for pilot and feasibility trials being undertaken in the United Kingdom registered in the United Kingdom Clinical Research Network database. BMC Med Res Methodol.

[17] Szende A, Oppe M, Devlin N, editors. EQ-5D value sets: inventory, comparative review and user guide, vol. 2. New York: Springer Science & Business Media; 2007.

[18] Paterson K, Nicholls M, Bennell K, Bates D. Intra-articular injection of photo-activated platelet-rich plasma in patients with knee osteoarthritis: a double-blind, randomized controlled pilot study. BMC Musculoskelet Disord. 2016;17:67. 10.1186/s12891-016-0920-3 PMC4748460 26861957

[19] Mustafa A, Mallen CDM, Murphy AWM, Glynn LG (2017). The effectiveness and safety of platelet-rich plasma intra-articular injections in the treatment of knee osteoarthritis: a systematic review and meta-analysis of randomised controlled trials.

[20] Shen L, Yuan T, Chen S, Xie X, Zhang C (2017). The temporal effect of platelet-rich plasma on pain and physical function in the treatment of knee osteoarthritis: systematic review and meta-analysis of randomized controlled trials. J Orthop Surg Res.

[21] Cerza F, Carni S, Carcangiu A, Di Vavo I, Schiavilla V, Pecora A (2012). Comparison between hyaluronic acid and platelet-rich plasma, intra-articular infiltration in the treatment of gonarthrosis. Am J Sports Med.

[22] Simental-Mendía M, Vílchez-Cavazos JF, Peña-Martínez VM, Said-Fernández S, Lara-Arias J, Martínez-Rodríguez HG (2016). Leukocyte-poor platelet-rich plasma is more effective than the conventional therapy with acetaminophen for the treatment of early knee osteoarthritis. Arch Orthop Trauma Surg.

[23] DeLong JM, Russell RP, Mazzocca AD (2012). Platelet-rich plasma: the PAW classification system. Arthroscopy.

[24] Jang S, Kim J, Cha S (2013). Platelet-rich plasma (PRP) injections as an effective treatment for early osteoarthritis. Eur J Orthop Surg Traumatol.

